# Hikikomori Phenomenon in East Asia: Regional Perspectives, Challenges, and Opportunities for Social Health Agencies

**DOI:** 10.3389/fpsyt.2019.00512

**Published:** 2019-07-23

**Authors:** John Chee Meng Wong, Michelle Jing Si Wan, Leoniek Kroneman, Takahiro A. Kato, T. Wing Lo, Paul Wai-Ching Wong, Gloria Hongyee Chan

**Affiliations:** ^1^Department of Psychological Medicine, National University Health Systems, Singapore, Singapore; ^2^NUS Mind-Science Centre, Department of Psychological Medicine, Yong Loo Lin School of Medicine, National University of Singapore, Singapore, Singapore; ^3^NUS Mind-Science Centre, Department of Psychological Medicine, Yong Loo Lin School of Medicine National University of Singapore, Singapore, Singapore; ^4^Department of Neuropsychiatry, Graduate School of Medical Sciences, Kyushu University, Fukuoka, Japan; ^5^Department of Applied Social Sciences, City University of Hong Kong, Kowloon, Hong Kong; ^6^Department of Social Work and Social Administration, Faculty of Social Sciences, The University of Hong Kong, Hong Kong, Hong Kong; ^7^Department of Social and Behavioural Sciences, Caritas Institute of Higher Education, Kowloon, Hong Kong

**Keywords:** hikikomori, social withdrawal, youth, Asia, challenges, opportunities

## Abstract

Hikikomori, which originated in Japan, refers to the condition where youths withdraw into the home and do not participate in society for an extended period of time. Recent updates on hikikomori presentation within the region were exchanged at a Hikikomori Round Table and Regional Symposium (HRTRS) discussion late 2017, leading to this perspective paper. Hikikomori presents as an overall homogeneous construct, while diversity in clinical presentation exists across East Asian countries. We examined the various presentations, risk factors, theoretical frameworks, and classification issues about hikikomori. In particular, specific risk factors have emerged to some degree across the region, while some are more locale specific. We propose that hikikomori youths have differential onset and developmental patterns, potentially resulting in heterogeneous presentation. We briefly summarized existing interventions in the East Asian region. Intervention strategies need to be tailored to different subtypes. A multicomponent approach would address complexity, multifactorial onset, and development of the condition. The HRTRS presented to participating countries the opportunity to collectively work toward a more universal definition of the hikikomori condition and explored innovative ways to shape existing service structures. Opportunities for participating countries described pertain to early detection of cases, adoption of assessment tools, and improved intervention services.

## Introduction

Youths who experience prolonged social withdrawal and retreat into homebound states have been referred to as hikikomori (Japan), hidden youth (China, Hong Kong, and Singapore), or socially withdrawn youth (South Korea and Hong Kong). They cause significant social and health concerns, with associated intervention complexities and poorly understood psychopathology, inviting controversy among social work agencies and policy makers (1).

At the Hikikomori Round Table and Regional Symposium (HRTRS) hosted by the National University of Singapore in November 2017, researchers and clinicians from Japan, Hong Kong, South Korea, China, and Singapore convened to share findings toward progressive understanding of the phenomenon.

The HRTRS aimed to describe varying cultural and regional presentations of hikikomori, highlight evidence-based interventions to map out a consensus toward clinical definition, and discuss appropriate sociopolitical responses for the East Asian countries represented. Although the condition has been ubiquitously reported globally, including several European countries, India, and the US (2–6), this article focuses on East Asia where the phenomenon is better differentiated and described. The present article aims to overview similarities and differences in identification, intervention, and support strategies to summarize opportunities in the region.

## Prevalence

Prevalence estimates are only available for Japan and Hong Kong (7, 8). For Japan, the lifetime prevalence rate of social withdrawal of 6 months or more was 1.2% (based on face-to-face survey), while for Hong Kong, it was 1.9% (based on telephone survey). Some argue that the hikikomori condition is becoming more prevalent. If so, it is not known whether this surge is due to increased awareness, improved identification of cases, or causes such as financial circumstance or increased internet usage. In particular, increased internet usage, online gaming, and consequently, the rise of internet-associated addictions may play an important part in the rising rates of social withdrawal (9). Online food delivery and shopping platforms that provide resources and services to the doorstep may further facilitate social withdrawal and disengagement.

## Clinical Presentation

During the HRTRS, a broad definition was used to accommodate regional differences. Hikikomori is a condition where socially withdrawn youths aged 12–30 years isolated at home for 3–6 months or more, may experience distress, impaired daily functioning, or psychiatric comorbidity. This definition implies that not all those with hikikomori are dysfunctional, and not all those with hikikomori perceive themselves as being dysfunctional. Existing assessment tools include screening questionnaires, such as a self-report Hikikomori screening questionnaire (HQ-25) validated in a Japanese sample (10), and a proposed diagnostic interview tool (6). In addition, research has included social interaction models and biomarkers (11, 12), tools not yet applicable to clinical assessments in distinguishing subtypes of hikikomori.

Hikikomori presents with different stage, duration, and degree of social withdrawal that may be due to differential developmental course of the condition. A Hong Kong study showed that self-reports of self-efficacy, self-esteem, subcultural identity, and empowerment were initially low in youths with 3 to 24 months of social withdrawal but improved beyond 24 months. This positive trend may be due to formation of a subcultural identity within the online community that allowed youths to realize their full potential. Moreover, prolonged social withdrawal was associated with improved physical, social, and psychological quality of life over time (13). Retrospective viewpoints of formerly withdrawn youth suggest that initial phases of social withdrawal may confer a private status that blinds them to the dangers of social skill deterioration and increased apathy, submersed in this state over time (14). These findings await replication in other regions. A cross-national study of 36 socially withdrawn males with an average duration of 2.1 years showed high levels of loneliness and moderate functional impairment (6), but the sample size did not allow for analyses by duration of social withdrawal. In addition, youths often show fluctuations in the degree of withdrawal in all regions (15). This varies from exclusive seclusion in one’s bedroom, to youths with no contact with others outside the nuclear family, to youths who infrequently leave home for sustenance.

Variance in duration and form of social withdrawal moderate the corresponding impact on the individual, family, and society. While some hikikomori may not experience a decrease in quality of life, negative behavioral and social consequences may arise from complications associated with a sedentary lifestyle. In Asian societies that emphasize individual industrialism and regular routines, disrupted dietary routines and sleep diurnal reversal patterns are frowned upon, compounded by negative impacts on physical health (12). Adverse social impacts on families include social distancing between family members, escalating to marital and parent–child conflict, sibling rivalry, loss of youth’s potential earnings, and depleting parents’ savings. Socioeconomic costs include reduced available human capital and possibly negative impacts on population growth.

## Typologies

Hikikomori carries high psychiatric comorbidity across Asian populations. A Japanese study showed 80% of help-seeking hikikomori met the criteria for a *Diagnostic and Statistical Manual of Mental Disorders, Fourth Edition, Text Revision* diagnosis (16), including avoidant personality, social anxiety disorder, and major depression. In addition, autistic spectrum disorders and prodromal states of schizophrenia may also demonstrate overlapping symptomatology (3, 6).

In order to further our understanding of the hikikomori phenomenon, a typology distinguishing primary versus secondary hikikomori has been proposed. Suwa and Suzuki (17) and Li and Wong (4) propose a distinction between primary versus secondary hikikomori. Primary hikikomori has no evident psychiatric disorder, while secondary hikikomori has social withdrawal attributable to the presence of psychiatric disorders. This classification of hikikomori is theoretical, while there is little empirical evidence for such a distinction (18). Existing literature indicates substantial numbers of secondary hikikomori in the respective studied populations. However, this may be ascribed to the fact that mental health and social services remain their main receptor platforms and treatment centers. In contrast, primary hikikomori may chiefly be identified through novel research studies designed to capture this elusive group. Furthermore, the primary hikikomori concept remains poorly defined and unstandardized across studies and countries. For instance, all socially withdrawn youths in South Korea are considered primary hikikomori, while Japan includes those with or without psychiatric comorbidities. This alludes to directional uncertainty on whether prolonged social withdrawal is caused by, correlated with, or causes psychiatric disorders.

Another theoretical framework describes socially withdrawn youths as a function of different interactions between individual, family, school, and social factors (4) *via* different types of social withdrawal processes. Some youths may lack autonomy due to overprotective parenting and financial overdependence. Others may experience existential uncertainty due to discrepancy between parental expectations for achievement and their prospective outcomes. However, no studies to date have examined these underlying mechanisms.

## Risk Factors

The best-known risk factors for hikikomori are the presence of a psychiatric disorder, developmental disorder, substance-related or behavioral addictive disorders (including Internet and gaming misuse), and poor psychosocial contexts (5, 16, 19). Various risk factors that emerge from the literature are universal among the East Asian regions, including male gender, insecure attachment (20), and psychiatric conditions. Conversely, other risk factors achieve less consensus. Studies on clinically detected hikikomori in Japan, for example, demonstrate that high educational status of families, especially fathers, is reported to increase the risk of hikikomori (21). However, it has been suggested that these studies of clinically detected hikikomori may not represent hikikomori receiving assistance from nonprofit organizations or no assistance at all (22). Hikikomori in Hong Kong detected through social service platforms, for instance, emerges in diversified contexts including low socioeconomic status (SES) families with single parents. However, 80% of cases from Hong Kong emerge from middle to high SES families, mirroring trends observed in Japan.

In countries with high costs of living such as Hong Kong and Singapore, a family with high SES may accommodate financial burden of the hikikomori individual. For anomalies with low SES, several clinicians at the HRTRS anecdotally hypothesized that they may possess specific attributes, such as high cognitive function or remote earning capabilities. Furthermore, low familial support and maternal mental disorder were also positively associated with hikikomori (21).

Dependent behavior described as “amae” in Japanese parent–child relationships has been hypothesized to play a role in developing social withdrawal (23) by normalizing and encouraging the acceptance of their socially withdrawn child (HRTRS) (24) staying at home. These mechanisms await further investigation in the various regions. East Asian parenting styles tend to embody the mother–child relationship as interdependent cooperation, in contrast to the focus on evolving independence and autonomy in Western regions (25). In Korea, the youth culture incorporates a strong sense of cohesion and engagement through social gatherings. When individuals are isolated for more than 6 months, it is perceived as a sign of mental illness. Lastly, preliminary studies in Japan and Hong Kong have begun investigating physiological biomarkers and physical health parameters, respectively, that may eventually contribute to early identification (11, 12).

Furthermore, studies point to the overlap between social withdrawal and Internet and gaming addiction [e.g. Refs. (18, 26)]. Benarous and colleagues, for instance, describe how avoidance and social withdrawal as a persistent maladaptive reaction in a patient with an anxious–avoidant insecure attachment plays a key role in the emergence and persistence of Internet gaming disorder. Lastly, the stigma surrounding mental illness diagnoses may result in preference for hikikomori labeling to mask psychiatric conditions.

**Table 1** shows inter-regional presentations of the hikikomori phenomenon, elucidating heterogeneity across regions. The infrastructure to address social or mental health issues determines main modes of identification for the autonomous regions and, in turn, the locale-specific hallmarks of the hikikomori condition. However, regardless of the identification method, hikikomori youths are a heterogeneous population due to differing causes unique to the specific youth.

**Table 1:** Cross-regional presentation of hikikomori phenomenon.

Country	Japan	Hong Kong	Singapore	Korea	China
Construct	•Hikikomori as a possible psychiatric condition	•Hikikomori as hidden youth with Internet/gaming addiction	•Hikikomori as socially withdrawn youth with school nonattendance, poor academic performance, and adjustment issues with peers	•Hikikomori as youth with Internet/gaming addiction	•Hikikomori as unemployed, unproductive youth members of society
Leading social health service providers	•Fukuoka Hikikomori Support Center •Mental Health clinics •Academic Research Centre for Hikikomori (Kyushu University Hospital - Hikikomori Support/Research to collect data, clarify biopsychosocial cause/ Intervention/Networking)	•Social service centers (CRYOUT)	•Mental health services (REACH West, NAMS) •Social services (TOUCH Cyber Wellness, MeToYou)	•Mental health hospitals •Social service centers •Internet addiction prevention centers	•Social service centers •Mental health system/centers
Available interventions	•Family Supports •Telephone and Direct Counselling •Use of HQ-25 as an evaluation and diagnostic assessment tool •Group Approach	•CRYOUT online gaming platform to identify hikikomori youths •Animal-assisted therapy •Social service conducts home visits. •Socialization, job training,and skills training	•Primary mental health system and school counseling services identify cases •Referred to mental health clinics or social services for intervention.	•School provides surveillance for social withdrawal among nonattendees •Use of social withdrawn youth questionnaire •Therapeutic programs include: •Internet addiction prevention, cyber counseling, home visitation, art therapy, case study	•Social work intervention: interviews, family involvement, reintegration with peers and society, skill building •Youth unemployment strategies over outreach to hikikomori.
Key research findings and associated risk factors	•Personality Types (Avoidant and Schizophrenic Personality) •Biomarkers (Low Uric Acid (males), Low High-Density Lipoprotein cholesterol (HDL-C) (females))	•Internet subcultural engagement, online network •Spiritual over material satisfaction •Sociodemographic differences in gender, age, education level, employment status, monthly income	•Gaming addiction •Cyber addiction •School nonattendance •Conscript military service avoidance •Psychological disorders (social anxiety disorder, autism spectrum disorder) •Subculture engagement (Otaku-like, counterculture)	•Cyber addiction •Social violence and bullying in schools •Alcohol abuse Internalizing, ruminative thoughts	•Social anxiety •Cyber addiction •Youth unemployment (oversaturation of university graduates) •Youth lacking financial independence
Next step(s) planned	•Develop assessment systems based on case presentation and biomarkers •Study “Modern-type depression” as a potential gateway disorder hence intervention target for Hikikomori.	•Secure funding to build services for hikikomori population •Adopt snowball sampling method to identify hikikomori related to Internet addiction youths.	•Increase awareness in early detection and treatment among professionals •Conduct focus group with hikikomori youth and their parents, to scope services •Develop novel outreach and composite intervention program •Provide specialized training for house visitation experts to handle comorbid presentations.	•Solicit inter-ministry support in funding, care policy, and program implementation. •Provide training for social workers to recognize hikikomori characteristics •Establish specialized hikikomori service centers.	•Conduct questionnaire survey with psychiatrists, psychologists, social workers •Conduct qualitative interviews with hidden youths •Establish an integrated intervention model •Research into mental health de-stigmatization •Uncover hidden societal impact and costs.

## Therapeutic and Preventive Interventions

Various healthcare interventions including family, individual, and group counseling or therapy, clinical psychological assessment, and medication or hospitalization exist for hikikomori in various countries. In addition, support and management services including group and interest activities, socialization and skills training, parenting advice, and community education may also be offered (27). Different interventions exist in disparate East Asian regions (refer to **Table 1**). These include home visits, befriending, psychodynamic therapy, parent support groups, Internet addiction programs, art therapy, cyber counseling, e-mentoring, family therapy, pharmacotherapy, animal-assisted therapy (AAT), and halfway homes. Specialized services catered to the hikikomori population exist in Japan and Hong Kong. Interventions are offered through specialized centers (Japan), general (psychiatric) hospitals (China and Singapore), schools (Singapore), government agencies (South Korea) or voluntary welfare organizations, and social agencies (Hong Kong, Singapore, and Japan). Interventions are often administered by social workers and mental health professionals. In China, apart from social work intervention (28, 29), ideological and moral education have also been advocated (30). Currently, referrals are based on condition presentation and location of identification. For instance, a socially withdrawn youth replying to a question in an online game receives cyber counseling, school nonattenders receive intervention through governmental agencies, and parents of socially withdrawn children mostly turn to psychiatric hospitals.

To date, published evidence on treatments for this group is scarce. In their review, Li and Wong (4) identified one empirical evaluation of an intervention specifically targeted at the hikikomori condition, involving a home visitation program conducted in Korea (19). More recently, Wong and colleagues (31) examined the effectiveness of a multicomponent program with animal-assisted therapy (AAT) for socially withdrawn youth in Hong Kong. Program evaluation indicated enhanced self-esteem and perceived employability, and reduced social anxiety. The AAT component did not significantly add to program outcomes but appeared effective in promoting enrolment. During the HRTRS, various strategies to attract and incentivize youth into accepting social and therapeutic interventions were discussed. These include online treatments, potentially incorporated into online gaming, and messaging platforms in Hong Kong’s programs. However, the efficacy of these pilot interventions has yet to be assessed. In a cross-national study of 36 socially withdrawn males, a consistent preference for treatment delivered in person over tele-psychiatry was expressed (6). Although social media advertising has demonstrated nominal capacity to detect hikikomori (32), the potential for effective online interventions to bridge the therapeutic gap remains unclear.

In line with other mental health conditions and drawing from the various presentations, intervention strategies evidently need to be tailored to heterogeneous hikikomori subtypes. A multicomponent approach would address complexity, multifactorial onset, and development of the condition. This multicomponent approach should always include youth empowerment and family interventions, whereas AAT should be second-line treatment. For instance, the most severe cases of exclusive seclusion at home may require intensive home visits, coupled with AAT. Alternatively, youths experiencing withdrawal as a transitory state may respond to interventions targeting school resumption and reemployment. In addition, interventions may differ depending on withdrawal duration. Earlier intervention is advantageous to directly address youths with impaired daily functioning. The optimal time frame for intervention appears to be after 3 months of withdrawal, before youths are officially characterized as hikikomori (withdrawal >6 months). At this interval, the joys of regained autonomy have faded, and youths begin to realize the downside but have yet to regain renewed “self-efficacy” and a new sense of hikikomori identity cultivated by a prolonged state of social withdrawal—typically after 2 years.

## Future Directions: Challenges and Opportunities

Challenges across countries concern the difficulty of case detection due to its hidden nature (32) and that youths may not pathologize this state. Another closely related challenge relates to motivating hikikomori youth to access services or treatment and to return to society. The fragmented knowledge of the condition also hampers optimal intervention. The heterogeneity in presentation, developmental pathways, risk and protective factors, and underlying mechanisms, within regions and across regions, have led to a wide range of services currently being offered, often of unknown efficacy.

The components of challenges at the societal or country level include recognizing burden of care on family and society and the role of authorities or policy makers. As the individual is nested in an ecological social system, the involvement of peers and family is essential to scaffold intervention. Authorities and policy makers may prioritize service planning and implementation by reinforcing case detection by effective gatekeepers. State policy and funding should also focus on awareness, advocacy, and fostering interorganizational collaboration.

The HRTRS presented an opportunity to collectively work toward a more universal definition of the hikikomori condition and explore innovative ways to shape existing service structures. These opportunities pertain to early detection of cases, adoption of assessment tools, and improved intervention services.

Development of opportunities is critical for early detection in the respective regions, through evolving the existing service structures to fit the needs of their youths. For example, primary and secondary school attendance is compulsory and tracked in Singapore. Prolonged absence from school without medical reason could trigger a home visit by a trained social worker. In Hong Kong, it may be helpful to raise awareness to recognize hikikomori symptoms among general practitioners, teachers, and parents, since hikikomori is currently mainly detected through online gaming platforms.

Next, standardization of screening and assessment tools would align primary evaluation within a validated diagnostic framework. We recommend to adopt the HQ-25 screening instrument, as it is theoretically grounded, easy to administer, and shows encouraging psychometric properties (10). We propose that a common method of identification would allow construction of a pathway to justify combinations of recommended interventions. These interventions would directly target affirmed symptomology and causes specific to the hikikomori individual.

As hikikomori is a multifaceted condition, an intervention model that integrates various possible factors affecting the individual could structure and direct intervention. Each country has differing approaches to case identification but may additionally adopt aspects of intervention from other regions to construct a complete biopsychosocial intervention. For example, Singapore may consider adapting AAT into their programs for hikikomori with low motivation and severe withdrawal. For Japan, which has strengths in biological identification and psychiatric and social services, they may consider working toward a more integrated and unified approach. Through a shared information network on research protocol and standardized outcome measurements, different countries may create their own integrated intervention model specific to local needs.

Furthermore, it may be beneficial for regions to examine innovative treatment approaches. The use of augmented reality games to draw socially withdrawn youths out of their rooms and into the real world (33, 34) has recently been suggested. One potential development may include introducing gaming stations at therapy centers and devising activities that encourage offline cooperation and interaction. Another potential avenue pertains to integrating therapeutic gaming with exposure therapy strategies by introducing hikikomori to virtual reality experiences, then shared virtual reality environments, before resocialization into society (9).

The lack of large-scale standardized research has previously been highlighted by multiple authors (4, 35). Future studies should include structured psychiatric assessments to investigate primary hikikomori and associated psychosocial comorbidities to better delineate the cause–effect relationship and intervention touch points for improved outcomes. Furthermore, the debate underlying hikikomori classification is between one camp that contends that it deserves a stand-alone diagnosis in the *Diagnostic and Statistical Manual of Mental Disorders* versus another that argues hikikomori is simply a secondary effect of other psychiatric conditions. Select studies currently differentiate between onset of other psychiatric conditions before, during, and after hikikomori onset (7). This operational method could be used to differentiate primary and secondary hikikomori and align existing and future literature in streamlining manifestation of psychiatric comorbidity. This would better define case selection for cluster analysis of treatment outcomes and comparison across countries.

In addition, the examination and detection of precursors in large-scale longitudinal epidemiological studies will further enhance early intervention opportunities to prevent withdrawal or limit withdrawal duration. Future studies should also examine Internet and gaming addictions in relation to the hikikomori condition. These collective efforts will further our understanding of this complex phenomenon and consequently improve services provided to prevent or mitigate youth from prolonged social withdrawal.

## Author Contributions

JW and MW organized the HRTRS and collated all discussions. MW and LK wrote the manuscript. TK, PW, WL, and GC contributed their insights to the HRTRS and the manuscript. All authors contributed to manuscript revision and read and approved the submitted version.

## Funding

The Round Table and Regional Symposium have been partly funded by the NUS Mind Science Center, National University of Singapore. No other sources of funding have been received for this event.

## Conflict of Interest Statement

The authors declare that the research was conducted in the absence of any commercial or financial relationships that could be construed as a potential conflict of interest.
